# How to Stop the Bleed: First Care Provider Model for Developing Public Trauma Response Beyond Basic Hemorrhage Control

**DOI:** 10.5811/westjem.2019.11.44887

**Published:** 2020-02-25

**Authors:** Joshua P. Bobko, Dylan J. Badin, Leila Danishgar, Kate Bayhan, Kevin J. Thompson, William J. Harris, R. Todd Baldridge, Gerald R. Fortuna

**Affiliations:** * Loma Linda University, Department of Emergency Medicine, Loma Linda, California; ‡ Dartmouth Geisel School of Medicine, Hanover, New Hampshire; § University of California, Irvine, Department of Emergency Medicine, Orange, California; ¶ First Care Provider Foundation, Corona del Mar, California; || California State University, Fullerton, Department of Health and Human Development, School of Nursing, Fullerton, California; # Citrus College, Department of Health Science, Glendora, California; ** Washington University in St. Louis, Department of Surgery, St. Louis, Missouri

## Abstract

**Introduction:**

Since 2013, the First Care Provider (FCP) model has successfully educated the non-medical population on how to recognize life-threatening injuries and perform interventions recommended by the Committee for Tactical Emergency Casualty Care (C-TECC) and the Hartford Consensus in the disaster setting. Recent programs, such as the federal “Stop The Bleed” campaign, have placed the emphasis of public training on hemorrhage control. However, recent attacks demonstrate that access to wounded, recognition of injury, and rapid evacuation are equally as important as hemorrhage control in minimizing mortality. To date, no training programs have produced a validated study with regard to training a community population in these necessary principles of disaster response.

**Methods:**

In our study, we created a reproducible community training model for implementation into prehospital systems. Two matched demographic groups were chosen and divided into “trained” and “untrained” groups. The trained group was taught the FCP curriculum, which the Department of Homeland Security recognizes as a Stop the Bleed program, while the untrained group received no instruction. Both groups then participated in a simulated mass casualty event, which required evaluation of multiple victims with varying degree of injury, particularly a patient with an arterial bleed and a patient with an airway obstruction.

**Results:**

The objective measures in comparing the two groups were the time elapse until their first action was taken (T1A) and time to their solution of the simulation (TtS). We compared their times using one-sided t-test to demonstrate their responses were not due to chance alone. At the arterial bleed simulation, the T1A for the trained and untrained groups, respectively, were 34.75 seconds and 111 seconds (p-value = .1064), while the TtS were 3 minutes and 33 seconds in the trained group and eight minutes in the untrained groups (physiologic cutoff) (p-value = .0014). At the airway obstruction simulation, the T1A for the trained and untrained groups, respectively, were 20.5 seconds and 43 seconds (p-value = .1064), while the TtS were 32.6 seconds in the trained group and 7 minutes and 3 seconds in the untrained group (p-value = .0087). Simulation values for recently graduated nursing students and a local fire department engine company (emergency medical services [EMS]) were also given for reference. The trained group’s results mirrored times of EMS.

**Conclusion:**

This study demonstrates an effective training model to civilian trauma response, while adhering to established recommendations. We offer our model as a potential solution for accomplishing the Stop The Bleed mission while advancing the potential of public disaster response.

## INTRODUCTION

Active violence incidents continue to push the envelope of prehospital trauma care. The improvised explosive devices (IED) used in the 2013 Patriot’s Day bombing in Boston left three dead and 265 injured.^1^ While the attacks in Orlando, Dallas, and San Bernardino injured 152 and killed 68, these numbers could have been much higher if the IEDs in San Bernardino had performed as planned.^2^ As these attacks become more deadly and elaborate, so too must public preparation. Despite improved integration in active shooter incidents, first responders are challenged by caring for large numbers of victims within a “hot zone” where the threat is still ongoing. Despite our best efforts, victims of active shooter incidents face delays in receiving healthcare.^3^

Knowing that any delay in the treatment of trauma injuries can increase mortality, many agencies have made recommendations to include bystander involvement into the planning framework for both natural and man-made disasters.^4^,^5^ Since the First Care Provider (FCP) concept was proposed at the Medical Response to IED/Active Shooter Next Steps & Tactical Emergency Medical Services (TEMS) Standardization summit in 2014, there has been consensus among trauma providers and EMS systems that a community response is necessary. Following this meeting, the Hartford Consensus III documented the need for “empowering the public to provide emergency care” and recognizing hemorrhage control techniques.^6^

Concurrently, the Committee for Tactical Emergency Casualty Care (C-TECC) created a working group to research the evidence to support the education of non-medical providers.^7^,^8^,^9^ In 2015, the FCP white paper described the systemic requirements for community empowerment.^10^ Most recently, in 2015, the Presidential Policy Directive on Preparedness and the Department of Homeland Security (DHS) announced the “Stop the Bleed” campaign, which recognized the need for early hemorrhage control through the widespread use of tourniquets.^11^

Events in Boston and as Vegas, in particular, reveal that access to the wounded, recognition of significant injury, and rapid evacuation to medical care is at least equally important as immediate hemorrhage control.^12^,^13^ A recent study published in the *Journal of Trauma* proposed a framework for how these concepts could be incorporated by smaller agencies.^14^ We propose that our FCP training, which is recognized by the DHS as a Stop the Bleed program, is an efficient and effective means of educating the civilian public to recognize trauma, identify life-threatening physiology, and empower them with the tools to prevent traumatic mortality.

## MATERIALS AND METHODS

Our hypothesis in initiating the study was that by providing non-medical lay public with a structured public educational model based on existing C-TECC and Hartford Consensus recommendations and as outlined in the FCP white paper, civilians would be able to successfully assess and treat the most common causes of preventable death during disaster scenarios.

**Population Health Research Capsule**

What do we already know about this issue?

Despite growing worldwide momentum for “Stop the Bleed” interventions by civilians, no studies to date have validated the effectiveness of available curricula.

What was the research question?

Can a curriculum be shown to improve both medical skills and recognition of life-threatening injury?

What was the major finding of the study?

Laypersons trained as First Care Providers responded to trauma faster than nursing graduates or untrained public.

How does this improve population health?

We demonstrate an effective, reproducible model for improving disaster resilience by developing public trauma response beyond basic hemorrhage control.

### Participant Selection

Participants for this study were canvassed as volunteers through the city of Westminster, California, with the goal of representing a cross-sectional demographic of the local population. The 75 volunteers included recent nursing graduates and undergraduate nursing students, local teachers, city employees, private security personnel, and high school students. A total of 51 participants took part in the exercise. Prior to the evaluation phase of the program, the volunteers were then assigned into “trained” and “untrained” groups. Newly graduated nurses with a Bachelor of Science with a major in nursing (BSN) degree served as the control for recent, medically “trained” individuals without FCP training. They were included to determine whether any trauma response had been incorporated into their recent nursing curriculum. A local fire department engine company was used as a first responder (EMS) baseline for any natural or man-made disaster.

### Training

In conjunction with an ongoing disaster effort piloted by the city of Westminster (CA), each of the trained groups participated in the four-hour FCP curriculum, which is recognized by the DHS as a Stop the Bleed program. This interactive lecture familiarized students with the DHS “Run, Hide, Fight” curriculum, activating the emergency response system, applying the TECC medical guidelines for civilians, and familiarized them with trauma equipment. The training seminars were conducted six weeks prior to the simulation. Prior to participation in the natural disaster simulation, all participants took a pre-test with 14 questions. Participants first self-identified their level of training. The remaining 14 questions were designed to assess the participant’s understanding of general trauma and current level of comfort and preparedness, with and without training.

### Simulation and Grading

To simulate a disaster, the event was held in an open storefront at the local mall during daytime operations. To ensure reproducibility, each group received a scripted overview detailing the exercise scenario: a large earthquake. The briefing included rules of engagement, set expectations, and defined objectives (Appendix 1). The room was arranged to simulate a major earthquake with debris strewn about and lighting problems. The subjects were assessed in groups, to both maintain realism as well as the integrity of each group’s interventions.

Each group encountered the same four victims. Victim 1 was deceased with a closed head injury. This injury pattern ensured that trainees had been adequately trained on assessment of life or death. Victim 2 had a simulated arterial bleed and open chest wound. This pattern was selected to evaluate prioritization of bleeding control in a complex wounding pattern. Victim 3 was unconscious but breathing, to assess subject’s ability to maintain airway patency while assisting other first care providers. Finally, Victim 4 had only superficial injuries. This use of a “distractor” was meant to challenge the subject’s ability to perform assessments on animated patients and prioritize more severe injuries. Again, to ensure reproducibility, victims received scripted information including type of injury and appropriate interaction with subjects.

The participants were evaluated on two criteria: time to first action (T1A) and time to solution (TtS). T1A was identified as a surrogate for recognition of a preventable cause of death. This subjective marker recognized the participant’s first response, whether moving toward a victim, instructing others, calling 9-1-1, or retrieving a trauma kit. TtS was an objective marker that records a proper intervention on a preventable cause of death. This data was captured through redundant mechanisms. First, a time was digitally recorded by tactical operations manikins (TOMManikin models) donated by Innovative Tactical Training Solutions (ITTS) and operated by an ITTS professional representative. Additionally, each evaluator was given a standardized scoring sheet and assigned to only evaluate one “victim” (Appendix 2).

We did not limit the subject’s interaction with the victims, although a maximum “physiologic viability” time of eight minutes was recorded. This time was allotted to generously account for either exsanguination or fatal anoxic injury. Evaluators did not interact with the test subjects during the simulation.

### Analysis

We compared the trained and untrained groups using a one-sided t-test, with the test looking for “less.” This tests for “trained” having a smaller mean than “untrained.” The alternative hypothesis that we are rejecting is that the true difference in means is less than zero at a 95% confidence interval (CI).

## RESULTS

### Pre-Test Results

All participants were given a 14-question pretest. The questions were selected to provide insight into perceptions held by participants, and to focus on areas for instruction and barriers to retention. The following five questions demonstrate significant findings in the responses.

Question 1: “What is the number one cause of death in the US population ages 1–44?” The correct answer, “Trauma,” was appropriately identified by 85% of the trained participants, as opposed to only 15% of the untrained participants (Table 1). Also of note was the preponderance of security officers who answered cardiac arrest as the leading cause of death. This likely reflects conditioning of non-medical personnel by the training they receive (e.g., cardiopulmonary resuscitation [CPR] training).

Question 2: “What do you think is the standard response time for a medical emergency when 9-1-1 is called?” This question was answered correctly as 8–11 minutes by only 35% of the trained individuals and 11% of the untrained individuals (Table 2).^16^ A majority of participants believed the correct answer to be 5–7 minutes. This “public perception gap” may be propagated by the reported “successes” of the combined response in the Boston bombing and other recent terror incidents.^12^,^17^

To determine the mindset of course participants, Question 3 gave test subjects a range of options describing their primary concern following a disaster or emergency situation. The results show that “safety” was widely identified at 86% (Table 3). Interestingly, no participants listed treating other victims as their main concern. This result is intriguing because we see a natural response to find safety or shelter as the driving motivation. This facilitates education of the “Run, Hide, Fight” curriculum and allows a natural conduit to more complex discussions such as communicating with emergency dispatchers and providing medical care.

We also sought to evaluate common misconceptions regarding tourniquet use. Question 9 (Table 4) focused specifically on civilian application of a tourniquet to someone who is bleeding and asks whether the subject would remove it because of pain. The correct response is to reassure them and leave the tourniquet in place, as it could prevent the victim from exsanguination. All participants nearly unanimously identified this, with 88% responding correctly (Table 4). This finding encourages continued focus on hemorrhage control programs such as the federal Stop the Bleed campaign.

Finally, in order to understand the barriers to public implementation, the participants were asked what would prevent them from intervening on behalf of a victim following a disaster or emergency situation (Question 5). These groups were split across three answers: not knowing what to do (lack of education); uncertainty whether their assistance would make the victim worse (lack of understanding); and their concern for disease. Only two test participants identified litigation as a reason to not render aid in an emergency situation. This finding is open to interpretation, but appears to suggest that the overwhelming majority of people are willing to aid others in a disaster provided they have a framework for providing such care.

### Simulation Results

Current recommendations by the Hartford consensus and the TECC Committee suggest that the priorities of civilian care in a disaster situation should be focused on hemorrhage control, airway maintenance, and rapid extrication to medical facilities.^12^ Our study focused on the two objective medical interventions from these recommendations. For our results, we have included “trained” civilians with untrained civilians, and made comparison to an engine company first responders who are regarded as trained in disaster response, and new-graduate nurses (BSN graduates) who are regarded as individuals recently involved in standard healthcare curricula including CPR. Nursing undergraduate results were compiled with the “untrained” civilians.

### Time to First Action (T1A) - Arterial Bleed Station

**Figure 1:**
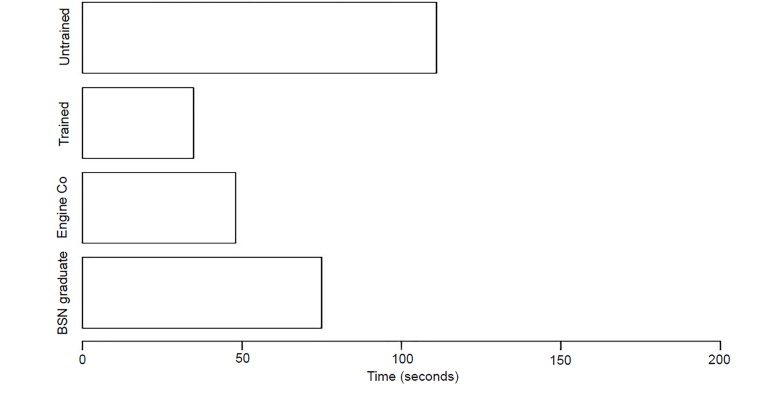
Time to first action of trained vs untrained groups in arterial hemorrhage control scenario, as well as of emergency medical services and healthcare (nursing graduates) professionals. *Engine Co*, fire department first responders; *BSN*, Bachelor of Science with a major in Nursing.

In our simulation, subjects were timed and their initial actions were monitored and recorded. When responding to the victim with an arterial bleed and open chest wound, the trained group performed their first action in an average time of 34.75 seconds, while the untrained group performed their first action with an average time of 111 seconds (p-value = .1064, CI (−∞, 47.15)). The engine company provided a first action time of 48 seconds. This served as a baseline for “First Responders” (Figure 1).

All trained group’s first action was to control the hemorrhage, either by direct pressure or through the use of a tourniquet. The untrained teachers and municipal employees did not treat this victim. The untrained security guards and students unsuccessfully attempted improvised tourniquets. It is worth noting that one of the untrained students was a former Junior Reserve Officer Training Corps (JROTC) candidate with previous tourniquet instruction. The T1A for nursing graduates (registered nurse (RN) or RN-eligible) was 75 seconds, with evaluation of the bleeding as their first action and direct pressure next. The nursing undergraduate students simply applied a non-occlusive compression wrap with a time of 60 seconds. Only one untrained students’ group and the engine company addressed the open chest wound, which was covered by debris.

### Time to First Action (T1A) - Airway Obstruction Station

**Figure 2:**
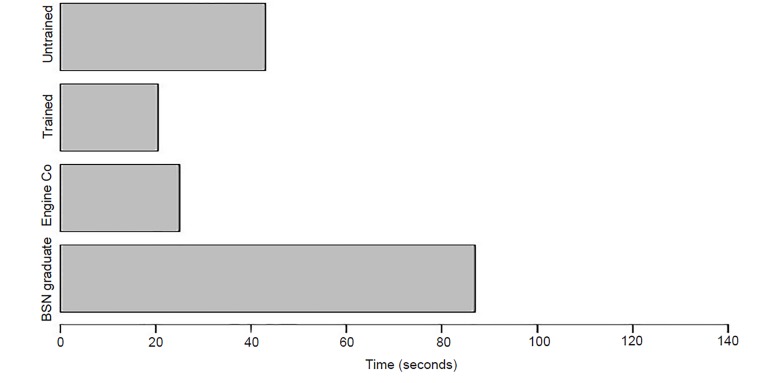
Time to First Action, of trained versus untrained groups in compromised airway scenario, as well as emergency medical services and health care professionals = nursing graduates. *Engine Co*, fire department first responders; *BSN*, Bachelor of Science with a major in Nursing.

The average T1A of the trained groups responding to the airway-compromised victim was 20.5 seconds, while the T1A of the untrained groups was 43 seconds, respectively (p-value = .0659, CI, −∞, 2.73524). The T1A for the trained groups was similar to that of the EMS baseline, which had a first time to action of 25 seconds. All trained groups placed the victims in the rescue position to maintain airway competency. The untrained city worker and teacher groups both placed the victim in an unsustainable position that compromised the airway immediately after their attempt at intervention. The EMS providers first performed a jaw thrust, and then instructed actor “bystanders” to maintain the position. After assessing the scenario, EMS returned to the “airway” victim and placed him in the rescue position. The RNs responded with a jaw thrust maneuver at 1 minute and 27 seconds, while the nursing undergraduate students performed CPR at 1 minute and 3 seconds (Figure 2).

### Time to Solution (TtS) - Arterial Bleed Station

**Figure 3:**
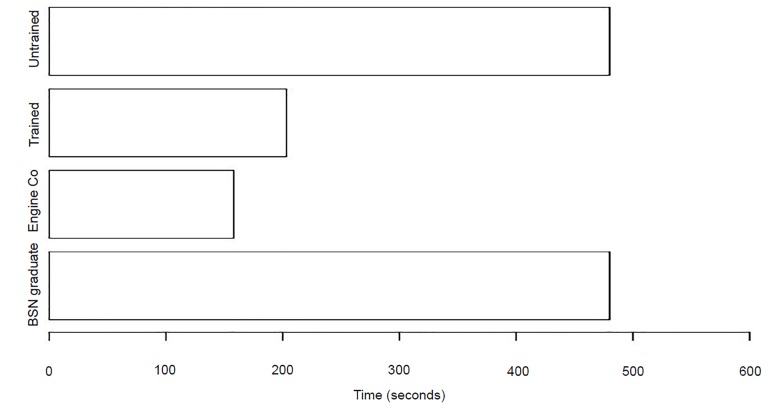
Time to Solution (TtS) of trained vs untrained groups in arterial hemorrhage control scenario, as well as emergency medical services and healthcare (nursing graduates) professionals (p-value = 0.001446, confidence interval [CI], −∞, −204.416). *Engine Co*, fire department first responders; *BSN*, Bachelor of Science with a major in Nursing.

Students were instructed that when treating the arterial bleeding victim, the appropriate action is to immediately apply direct pressure to the wound and/or apply a tourniquet to the affected extremity. With regard to treating the arterial bleeding, the trained groups had a significantly faster time than the untrained group when preventing exsanguination (p-value = 0.001446, CI, −∞, −204.416). The four trained groups had an average time to solution of 3 minutes and 33 seconds, while the four untrained groups were unable to arrive at a solution before the eight-minute physiologic cutoff. The average TtS of the trained groups approached that of our EMS baseline designated by the engine company first responders, who had an average time to solution of 2 minutes and 38 seconds (Figure 3).

### Time to Solution (TtS) - Airway Obstruction Station

**Figure 4:**
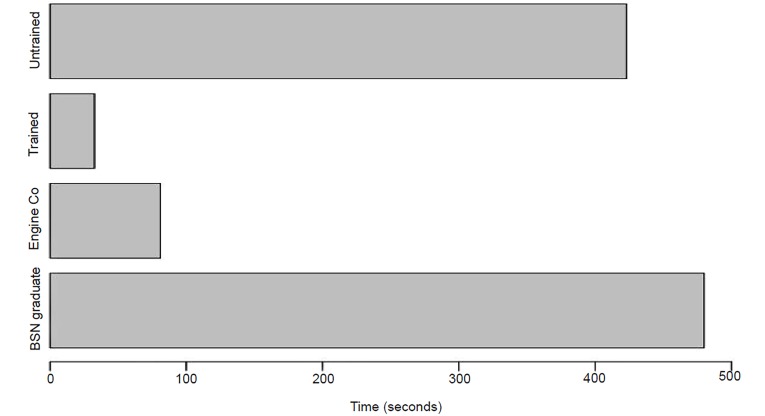
Time to Solution (TtS) of trained vs untrained groups in airway obstruction station, as well as emergency medical services and healthcare (nursing graduates) professionals (p-value = 0.008729, confidence interval [CI], [−∞, −191.5561]). *Engine Co*, fire department first responders; *BSN*, Bachelor of Science with a major in Nursing.

When assessing an unconscious victim, students were instructed to place the victim on his or her side to prevent airway aspiration or obstruction (e.g., Rescue or Recovery Position). The four trained groups had an average TtS of 32.6 seconds, while the four untrained groups had an average TtS of 7 minutes and 3 seconds. Once again, the trained groups performed a much more efficient TtS than that of the untrained group (p-value = 0.008729, CI, −∞, −191.5561). Only one untrained group was able to come to a solution before time expired (security officers). Once again, the trained groups’ average time to solution approximated that of the trained EMS professionals who had an average time to solution of 1 minute and 21 seconds (Figure 4).

## DISCUSSION

While the EMS response system in the United States has been evolving in reaction to active shooter events and disasters, there is still a notable delay.^13^ Because of the impact of such disasters, the push to incorporate civilian medical care is being viewed as a force-multiplier to existing response plans.^15^ While recommendations have been proposed to address this need in civilian action, no widespread implementation methods have been shown to be statistically beneficial.

Conversely, the FCP curriculum showed a threefold improvement in recognition and treatment of airway obstruction and control of arterial hemorrhage. There were additional positive outcomes associated with completion of the FCP curriculum. First, we can conclude that a concise, organized approach to disaster education stimulates independent thinking in the student population. While we used T1A as a marker for recognition of a preventable cause of death, it also served as an objective data point for action. In all cases, the trained groups moved with concise action when confronted with trauma victims, despite not meeting 95% CI. The TtS demonstrates that having a plan and knowing the basic signs to recognize victims leads to successful outcomes, even equal to those of EMS responders.

Furthermore, within these groups there was an observed willingness to lead the interaction with first responders. We propose two reasons for this observation. First, having an organized framework for responding to emergencies developed the students’ sense of control of a dynamic situation, which improved their ability to convey information to uniformed responders. Additionally, the guided medical training provided through the FCP curriculum lessened uncertainty regarding the care of those injured. The FCP curriculum enabled a technical foundation for decisive action, as well as a base for planning and a sense of control.

## LIMITATIONS

There are limitations to our study. Time constraints and the complexity of using an operational shopping center during working hours to stage a mock mass casualty incident contributed to the small number of test subjects in our sample set. The populations of both the trained and untrained volunteers represent another potential source of bias, although there were no exclusionary criteria for the two populations. Another source of potential bias was the use of the closest engine company as the “EMS/First Responder” control for our study. However, the consistency of the prehospital education curriculum was thought to negate any interdepartmental variation.^18^ Finally, the equipment used in the study was donated by Tactical Medical Solutions, Inc. Although the kit we used consisted of a windlass tourniquet, adhesive chest seals, gauze, and a trauma dressing, it is possible that brand familiarity may have affected outcomes.

Our preliminary study also revealed several potential areas for further investigation. The performance of the nursing graduates indicates a gap between policy recommendations and training curricula for our in-hospital healthcare providers.^19^ In addition, many agencies use the same criteria for tourniquet selection for public-access tourniquets as for first responders. Although there is widespread support encouraging civilian tourniquet use, there has yet to be a comparative analysis on the effectiveness of commercially available tourniquets applied by a purely civilian demographic in a stress-induced environment.^20^,^21^ It will be interesting to learn whether some requirements, such as one-handed application, are consistent in the civilian setting. Finally, while it has been demonstrated that children in sixth grade can effectively recognize cardiac arrest and use an automated external defibrillator, there is only anecdotal evidence that children can be effectively trained to recognize and intervene on the preventable causes of death in trauma.^22^ Statistical demonstration of effective education of this at-risk population would be critical.

## CONCLUSION

Our study demonstrates that it is possible to create an effective and retainable solution to disaster response to augment the first responder system while adhering to the recommendations of C-TECC, the Hartford Consensus, and the Department of Homeland Security. Further, because of its basis on well-recognized medical guidelines and ease of integration, the First Care Provider model provides an efficient and effective method for implementation of the federal government’s “Stop the Bleed” campaign, bridging the gap between theory and implementation. The FCP system can be integrated into local law enforcement and fire/EMS systems to reduce system reflex time to disaster and improve ground-zero time for response.

## Supplementary Information





## Figures and Tables

**Table 1:** Answers to Question 1 of the pre-test, organized by group number.

Group number	Group	Cardiac arrest	Trauma	Cancer	Medication OD
1	Nursing- grad	1	3		
2	Nursing- undergrad		4		
3	Teacher-trained		5		
4	Teacher-untrained				3
5	City-trained		5		1
6	City-untrained		2		3
7	Security-trained	1	2		
8	Security-untrained	4	1	1	
9	Students-trained		5		1
10	Students-untrained	3	1		1
11	Engine Co		4		

*OD*, overdose; *grad*, graduate; *undergrad*, undergraduate.

**Table 2:** Answers to Question 2 of the pre-test, organized by group number.

Group number	Group	2–4 min	5–7 min	8–11 min	12–15 min
1	Nursing-grad		3	1	
2	Nursing-undergrad		3		1
3	Teacher-trained		1	5	
4	Teacher-untrained		3		
5	City-trained	1	4		
6	City-untrained	1	4		
7	Security-trained		1	2	
8	Security-untrained		4	1	1
9	Students-trained	4	2		
10	Students-untrained	2	2	1	
11	Engine Co		1	3	

*min*, minutes; *grad*, graduate; *undergrad*, undergraduate.

**Table 3:** Answers to Question 3 of the pre-test, organized by group number.

Group number	Group	Call 911	Fleeing safety	Ensure safety	Treating victims
1	Nursing-grad			4	
2	Nursing-undergrad			4	
3	Teacher-trained			5	
4	Teacher-untrained			3	
5	City-trained			6	
6	City-untrained	3	1	2	
7	Security-trained			3	
8	Security-untrained	1		5	
9	Students-trained			6	
10	Students-untrained	1	1	3	
11	Engine Co			4	

*grad*, graduate; *undergrad*, undergraduate.

**Table 4:** Answers to Question 9 of the pre-test, organized by group number.

Group number	Group	Loosen the TQ	Remove the TQ	Reassure them	Tourniquets are an outdated means for hemorrhage control
1	Nursing-grad			2	2
2	Nursing-undergrad			4	
3	Teacher-trained			5	
4	Teacher-untrained			3	
5	City-trained			6	
6	City-untrained			5	
7	Security-trained			3	
8	Security-untrained			6	
9	Students-trained			6	
10	Students-untrained	1		3	1
11	Engine Co	1	1	2	

*TQ*, tourniquet; *grad*, graduate; *undergrad*, undergraduate.
