# Supplementary material for: How to Stop the Bleed: First Care Provider Model for Developing Public Trauma Response Beyond Basic Hemorrhage Control
Source: West J Emerg Med. 2020 Feb 25;21(2):365–73. doi: 10.5811/westjem.2019.11.44887 (PMC7081854; doi:10.5811/westjem.2019.11.44887)
Supplement: Supplementary file 1 [file wjem-21-365-s001.pdf]

EARTHQUAKE SCENARIO

SCENARIO BRIEFING (TO BE SHARED)

BACKGROUND:
You are having lunch at a mall when a magnitude 6.8 earthquake hits the metropolitan area. After the shaking has stopped, you notice that parts of the ceiling have collapsed, and visibility is difficult with back-up lighting. You can hear commotion and people calling for help. There are multiple obstacles and challenges that you will need to address. There may be victims requiring medical care, but all scenarios can be successfully addressed with materials present in the room. Proctors will wear colored vests and will not be allowed to interact with you until the scenario is complete.

ASSETS AVAILABLE:
-Anything available in the room
-Any prior training may be utilized

MODERATOR BRIEFING (CONFIDENTIAL)

CASUALTY ROSTER:
#1 Upper limb amputation from fallen structure
#2 Open chest wound from shattered glass + deep laceration to lower extremity
#3 Deceased
#4 Superficial wounds + Panic (ambulatory)
#5 Closed head injury, unconscious + supine with snoring respirations

MODERATOR INFORMATION:
- There will be a TRAUMEDIX™ kit mounted in the room
- Unstable structure becomes stable as long as casualty is extricated immediately
- Structure will collapse if extrication takes too long
- Victim #1 and #2 will die if hemorrhage is not controlled within 4 minutes
- Victim #5 must be put in recovery position for successful completion of scenario

OBJECTIVES:
1. Demonstrate appropriate “First Care” assessment of injured bystanders
