# Supplementary material for: How to Stop the Bleed: First Care Provider Model for Developing Public Trauma Response Beyond Basic Hemorrhage Control
Source: West J Emerg Med. 2020 Feb 25;21(2):365–73. doi: 10.5811/westjem.2019.11.44887 (PMC7081854; doi:10.5811/westjem.2019.11.44887)
Supplement: Supplementary file 2 [file wjem-21-365-s002.pdf]

FIRST CARE
PROVIDER

Mannequin Grading Sheet

Bleeding controlled?	Yes	No	Time:
Tourniquet applied?	Yes	No	Time:
TQ tight?	Yes	No	
Where was TQ applied?	High	3”-4” above	Over Joint
Extremity elevated?	Yes	No	
Pressure Dressing?	Yes	No	
Pt Bleed out at 3 minute mark?	Yes	No	
